# Molecular targeting of retinoic acid metabolism in neuroblastoma: the role of the CYP26 inhibitor R116010 *in vitro* and *in vivo*

**DOI:** 10.1038/sj.bjc.6603779

**Published:** 2007-05-08

**Authors:** J L Armstrong, G A Taylor, H D Thomas, A V Boddy, C P F Redfern, G J Veal

**Affiliations:** 1 Newcastle University, Northern Institute for Cancer Research, Paul O'Gorman Building, Medical School, Framlington Place, Newcastle Upon Tyne, NE2 4HH, UK

**Keywords:** retinoic acid, CYP26, R116010, neuroblastoma

## Abstract

Isomerisation to all-*trans*-retinoic acid (ATRA) is widely accepted as the key mechanism underlying the favourable clinical properties of 13-*cis*-retinoic acid (13cisRA). As intracellular metabolism of ATRA by CYP26 may result in clinical resistance to 13cisRA, an increase in efficacy may be achieved through modulation of this metabolic pathway. We have evaluated the effect of the CYP26 inhibitor R116010 on retinoid metabolism in neuroblastoma cell lines and a xenograft model. In neuroblastoma cells, which showed a high level of CYP26 induction in response to ATRA, R116010 selectively inhibited ATRA metabolism. In addition, siRNA-mediated knockdown of CYP26 selectively increased ATRA levels and the expression of retinoid-responsive marker genes was potentiated by R116010. Treatment of mice bearing SH-SY5Y xenografts with 13cisRA (100 mg kg^−1^) revealed substantial levels (16%) of intratumoral ATRA after 6 h, despite plasma ATRA levels representing only 1% total retinoids under these conditions. Co-administration of R116010 with 13cisRA in this mouse model resulted in significant increases in plasma ATRA and 13cisRA concentrations. Furthermore, R116010 induced significant decreases in levels of 4-oxo metabolites in hepatic tissue after co-administration with either ATRA or 13cisRA. These data suggest considerable potential for CYP26 inhibitors in the future treatment of neuroblastoma with 13cisRA.

Retinoic acid (RA) is an active metabolite of vitamin A, which plays an important role in cell growth and differentiation, and reverses malignant growth *in vitro* and *in vivo*. Retinoic acid and various synthetic analogues have proved useful agents in tumour therapy, particularly in the treatment of neuroblastoma, the commonest extracranial solid tumour of childhood (Reynolds *et al*, 2003). The long-term survival rates for stages 3 and 4 neuroblastoma remain low. In a setting of minimal residual disease, 13-*cis*-retinoic acid (13cisRA) improved event-free survival in advanced stage neuroblastoma patients (Matthay *et al*, 1999). Peak drug levels in patients receiving 13cisRA as high-dose pulse therapy after autologous bone marrow transplantation or non-myeloablative therapy were above the 5 *μ*M concentration effective against neuroblastoma cell lines *in vitro* (Villablanca *et al*, 1995). However, approximately 50% of patients develop resistance or are unresponsive to treatment. Potential approaches to improve therapy include the optimisation of pharmacokinetic properties of agents such as 13cisRA.

Retinoids exert their biological effects via nuclear receptors, the RA receptors (RARs: *α*, *β*, *γ*) and the retinoid X receptors (RXRs: *α*, *β*, *γ*) (Petkovich *et al*, 1987; Mangelsdorf *et al*, 1992). 13-*cis*-Retinoic acid has a much lower affinity for these receptors compared to its stereoisomers, all-*trans*-retinoic acid (ATRA) and 9-*cis*-retinoic acid (9cisRA), and is less active in transactivation assays (Idres *et al*, 2002). 13cisRA is, however, active in cultured cells and animal models at high doses (Tsukada *et al*, 2000; Ponthan *et al*, 2001; Veal *et al*, 2002). Although it is generally accepted that the effects of 13cisRA are mediated by isomerisation to the more transcriptionally active all-*trans* or 9-*cis* isomers (Armstrong *et al*, 2005a), this does not account for all pharmacological effects associated with the use of this retinoid (Blaner, 2001). In contrast to 13cisRA, unfavourable pharmacokinetic and toxicity profiles of ATRA and 9cisRA have limited their use in neuroblastoma therapy (Reynolds *et al*, 2003).

Cytochrome *P*450-mediated oxidation to 4-oxo-RA, 4-hydroxy-RA, 18-hydroxy-RA and 5,6-epoxy-RA represents the major route of retinoid catabolism (Marill *et al*, 2000, 2002). Three mammalian RA-inducible *P*450s have been characterised, termed *P*450RAIs or CYP26s (White *et al*, 1997, 2000). CYP26A1 and B1 exhibit a high degree of specificity for ATRA, while CYP26C1 also metabolises 9cisRA and is much less sensitive to the inhibitory effects of ketoconazole than A1 and B1 (Taimi *et al*, 2004). The induction of CYP26 in response to RA represents a developmentally important negative feedback loop controlling RA concentrations within cells, thereby limiting biological action. CYP26A1 mRNA induction has been reported in skin, intestinal, liver, APL and neuroblastoma cells treated with RA (Armstrong *et al*, 2005b; Ozpolat *et al*, 2005; Smith *et al*, 2006), and CYP26A1 protein is highly expressed in normal human skin (Heise *et al*, 2006).

Increased ATRA metabolism after prolonged ATRA treatment, both *in vitro* and *in vivo,* has led to the hypothesis that metabolism has a major role in the emergence of RA resistance (Muindi *et al*, 1992). Potentiating ATRA efficacy through inhibition of CYP26 is a novel approach and has led to the development of RA metabolism blocking agents (RAMBAs). Non-specific *P*450 inhibitors such as liarozole increase ATRA effectiveness *in vitro* and *in vivo* (Njar, 2002), but the use of liarozole in cancer patients has been limited by adverse side effects and only moderate ability to inhibit CYP26 (Njar *et al*, 2006).

A novel inhibitor of RA metabolism, R116010, enhances the biological activity of ATRA and exhibits antitumour activity as a single agent in a mouse mammary carcinoma model (Van Heusden *et al*, 2002). We have previously shown that R116010 inhibits ATRA metabolism in SH-SY5Y neuroblastoma cells and increases the pharmacological effects of both ATRA and 13cisRA (Armstrong *et al*, 2005b). The aim of this study was to measure the effect of R116010 on RA metabolism in a panel of neuroblastoma cell lines, which differ in their ability to upregulate CYP26 isoforms in response to RA, and to evaluate the effects of R116010 on RA metabolism in a neuroblastoma xenograft model.

## MATERIALS AND METHODS

### Chemicals

The RA isomers 13cisRA and ATRA were from Sigma (Poole, UK). 13-*cis*-4-oxo-RA (Ro 22-6595), all-*trans*-4-oxo-RA and acitretin (Ro 10-1670) were gifts from Hoffmann-La Roche. HPLC-grade solvents were from Fisher Scientific (Loughborough, UK). R116010 was kindly donated by Barrier Therapeutics Inc. (Princeton, NJ, USA).

### Cell lines

SH-SY5Y, SH-EP (N- and S-type cells respectively derived from the parental line SK-N-SH), LAN6, IMR-32, NGP and GIMEN neuroblastoma cell lines were cultured routinely in RPMI 1640 medium containing fetal calf serum (10%) and L-glutamine (2 mM). Cells were grown at 37°C in a humidified incubator containing 5% CO_2_.

### Mice

All *in vivo* experiments were approved by the relevant institutional animal welfare committees, and performed according to national law. Female athymic nude mice (CD-1 *nu/nu*, Charles River) used for antitumour studies were maintained and handled in isolators, under specific pathogen-free conditions, at 24°C with a 12 h light, 12 h dark cycle. Tumours were generated by implantation of SH-SY5Y cells subcutaneously (s.c.) into one flank of CD-1 nude mice (1 × 10^7^ cells per animal).

### Xenografting

Treatments began when the tumours were between 7 and 10 mm in length and breadth. Retinoids and R116010 were initially dissolved in DMSO before 10- or 20-fold dilutions in PBS, respectively. For pharmacokinetic studies, the animals were dosed with ATRA or 13cisRA (each 100 mg kg^−1^) orally for 0.5, 2 and 6 h. For metabolism studies, animals were treated with R116010 orally at doses of 1.25 or 2.5 mg kg^−1^ either immediately before the retinoid being studied, or up to three times (over 1–2 days) before dosing with the retinoid. At termination of experiments, animals were bled under terminal anaesthesia then killed by cervical dislocation. Plasma was immediately removed from the blood by centrifugation at 1000 **g** for 5 min and snap-frozen in liquid nitrogen in amber Eppendorfs. Tumour and liver samples were removed and snap-frozen, wrapped in foil, in liquid nitrogen.

### Retinoid treatment

13-*cis*-Retinoic acid and ATRA were dissolved in DMSO and diluted in cell culture medium to obtain final concentrations of 0.1–10 *μ*M. The final concentration of DMSO in all cases was <0.2%. All experiments were performed in dim light and tubes containing retinoids were wrapped in aluminium foil.

### Extraction of retinoids

Cell pellets obtained from retinoid incubations were re-suspended in 2 ml cell culture medium and disrupted by passing the samples several times through a syringe and hypodermic needle (25G × 1 inches, 0.5 mm × 25 mm; Terumo, Somerset, NJ, USA). Tumour and liver tissues were re-suspended in PBS and homogenised (T25 homogeniser, IKA Laboratories, Staufen, Germany). Retinoids were extracted as described previously (Veal *et al*, 2002; Armstrong *et al*, 2005b). Samples were reconstituted in 200 *μ*l mobile phase A (*n*-hexane:dichloromethane:propan-2-ol, 400 : 1 : 0.27) and analysed by normal-phase HPLC as described previously (Veal *et al*, 2002). For LC/MS analysis, samples were reconstituted in 200 *μ*l mobile phase A (50% acetonitrile, 50% (0.2%) acetic acid, v v^−1^).

### HPLC analysis

Quantification of retinoid levels was carried out by HPLC analysis using a Waters 2690 Separations Module and 996 Photodiode array detector (Waters Ltd, Elstree, UK) and Waters Millennium software for data acquisition. Cell volumes were determined as described previously (Veal *et al*, 2002), and intracellular RA concentrations were expressed in micromolars.

### LC/MS analysis

Resolution of retinoids was performed with a Luna C_18_(2) column (3 *μ*m, 50 mm × 2 mm) using a Perkin Elmer LC (Beaconsfield, UK) system, consisting of a vacuum degasser, two series 200 pumps, a thermostatically controlled series 200 autosampler and a Waters 2487 UV absorbance detector. Retinoic isomers and metabolites were separated by gradient reversed-phase chromatography. An injection volume of 20 *μ*l with a flow rate of 0.2 ml min^−1^ was used with mobile phase A (50% acetonitrile, 50% (0.2%) acetic acid, v v^−1^) and mobile phase B (acetonitrile, 0.1% acetic acid, v v^−1^). Linear gradients were employed between the specified times as follows: 0, 100% A; 5 min, 100% A; 15 min, 100% B; 20 min, 100% B; 23 min, 100% A; 35 min, 100% A. An Applied Biosystems (Warrington, UK) API-2000 LC/MS/MS triple Q (quadrupole) mass spectrometer with electrospray ionisation source, controlled by Analyst software, was operated in single quadrupole negative mode for the detection of compounds.

### RNA interference of CYP26A1 and B1

CYP26 short hairpin RNAs (shRNAs) were generated using the siSTRIKE U6 Hairpin Cloning System (Promega, Southampton, UK). The CYP26A1, B1 and Ctrl siRNA sequences were designed using Promega software. The cDNA target sequences were CYP26A1 5′-gcgcatcgagcagaacatt; CYP26B1 5′-gtcgcggagggagaagtat; and Ctrl 5′-gccccgcaattgagaaatg. Sense and antisense strands were annealed and ligated into the linearised psiSTRIKE Neomycin Vector. SH-SY5Y cells were transfected with psi-CYP26A1 or psi-CYP26B1 either singly or in combination, or with psi-Ctrl, in six-well plates (1 *μ*g each) or 75 cm^2^ flasks (8.9 *μ*g each) using FuGENE 6 reagent (Roche, Lewes, UK) according to the manufacturer's instructions. After a 6 h transfection, cells were treated with ATRA for 40 h and harvested for RNA isolation and HPLC analysis.

### Real-time PCR

RNA was reverse-transcribed using Promega's Reverse Transcription System according to the manufacturer's instructions, using random hexamer primers. Real-time PCR was performed on 20 ng cDNA using TaqMan gene expression products for human CYP26A1, CYP26B1, CRABPII, RAR*β* and MYCN in combination with the TaqMan Universal PCR master mix (Applied Biosystems, Warrington, UK). Appropriate controls for non-specific amplification and contamination were included. A GeneAmp 5700 Sequence Detection System was used for real-time PCR amplification. As internal standard, *β*-actin was measured simultaneously using the endogenous control assay provided by Applied Biosystems. PCR amplification procedures followed manufacturer instructions. Briefly, the thermocycling program consisted of one cycle at 50°C for 2 min followed by 95°C for 10 min and 40 cycles at 95°C (15 s) and 60°C (1 min). Standard curves were generated from serial dilutions of cDNA prepared from cells treated with ATRA (1 *μ*M) and the data were analysed using the GeneAmp Sequence Detection System software. CYP26C1 expression was analysed as described previously (Taimi *et al*, 2004).

### Statistical analysis

Analysis was performed on log-transformed data. General linear models (GLMs) and analysis of variance (ANOVA) were used to compare the effects of R116010 on retinoid concentrations in cell lines and tissues. Since dose (retinoid or R116010) is a quantitative factor, we have used linear contrasts in the GLM or ANOVA models (where the sum of contrast coefficients=0) to test for dose dependency between treatment and effect. Dunnett's test was used to compare treatment with control means. Degrees of freedom in statistical tests are given as subscripts to the test statistic. Statistical analyses were carried out using Systat, version 10 (SPSS Inc., Chicago, IL, USA) and SPSS release 11.0 (SPSS Inc.).

## RESULTS

### CYP26 isoform induction in neuroblastoma cell lines

**Figure 1:**
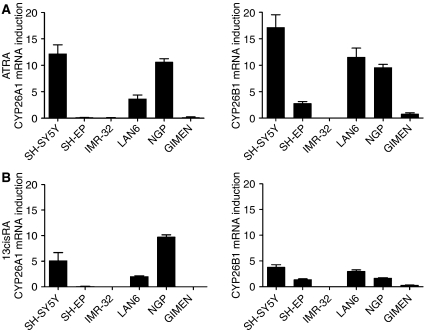
CYP26 induction in a panel of neuroblastoma cell lines. Reverse-transcribed RNA from neuroblastoma cells treated with 0.1 *μ*M ATRA (**A**) or 13cisRA (**B**) for 24 h was subjected to real-time PCR using TaqMan probes for CYP26A1, CYP26B1 and *β*-actin. Values are normalised for *β*-actin levels and expressed as fold increase relative to expression of CYP26 in SH-SY5Y cells treated with 0.01 *μ*M ATRA. Data are mean values±s.e.m. (*n*⩾3).

Since CYP26 isoforms were not detectable in cells in the absence of RA, the level of CYP26 induction in each cell line was assessed relative to CYP26 expression in SH-SY5Y cells treated with 0.01 *μ*M ATRA for 24 h. After incubation with 0.1 *μ*M ATRA (Figure 1A) CYP26A1 was induced to various extents in SH-SY5Y, LAN6 and NGP cells, while CYP26B1 was induced in all cell lines examined, except IMR-32. Incubation with 0.1 *μ*M 13cisRA (Figure 1B) produced a similar expression pattern, although the magnitude of response was lower compared to ATRA. CYP26C1 expression was not detected (by reverse transcription PCR) in any of the cell lines examined. These results show that CYP26A1 and B1 expression are very sensitive markers of retinoid response in neuroblastoma cell lines.

### R116010 increases retinoid response in neuroblastoma

**Figure 2:**
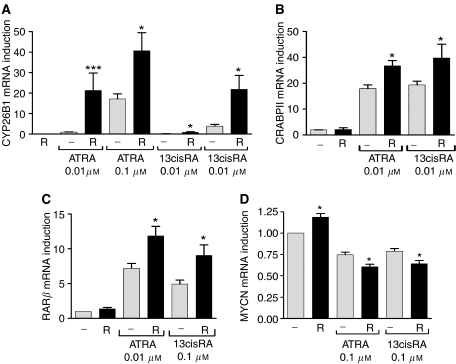
Effect of R116010 on retinoid response. SH-SY5Y cells were incubated with ATRA or 13cisRA (0.01 or 0.1 *μ*M) for 24 h in the absence or presence of R116010 (R, 1 *μ*M). Reverse-transcribed RNA from SH-SY5Y (**A**–**C**) or NGP (**D**) cells was subjected to real-time PCR using TaqMan probes for CRABPII, RAR*β*, CYP26 B1, MYCN and *β*-actin. Values are normalised for *β*-actin levels and expressed as fold increase relative to gene expression in control untreated cells (**B**–**D**), or to CYP26B1 expression in SH-SY5Y cells treated with 0.01 *μ*M ATRA (**A**). Data are mean values±s.e.m. (*n*⩾3). Statistical significance of gene expression in cells treated with RA in the presence of R116010 relative to those treated with RA alone is indicated by ^*^P<0.05, ^***^P<0.001.

To assess the effect of R116010 on retinoid response we analysed the expression of CYP26B1 and other retinoid responsive genes. CYP26B1, cellular RA-binding protein II (CRABP II), and RAR*β* expression were analysed in the retinoid-responsive SH-SY5Y cell line and MYCN expression analysed in the MYCN-amplified NGP cell line. Figure 2A shows that incubation of SH-SY5Y cells with R116010 alone (1 *μ*M) had no effect on CYP26B1 expression, but R116010 co-incubated with either ATRA or 13cisRA (0.01, 0.1 *μ*M) significantly increased CYP26B1 expression compared to ATRA or 13cisRA alone. There were also significant increases in both CRABP II and RAR*β* expression when SH-SY5Y cells were incubated with ATRA (0.01 *μ*M) or 13cisRA (0.1 *μ*M) in the presence of R116010 (1 *μ*M) compared to either retinoid alone (Figure 2B and C). R116010 alone resulted in a small but significant increase in MYCN expression in the MYCN-amplified NGP cell line. However, the downregulation of MYCN expression by ATRA or 13cisRA (0.1 *μ*M) was enhanced in the presence of R116010 (1 *μ*M) compared to either retinoid alone (Figure 2D). The magnitude of these responses was greatest with CYP26B1, demonstrating that CYP26 is a more responsive marker.

### Effect of R116010 on intracellular retinoid concentrations

**Figure 3:**
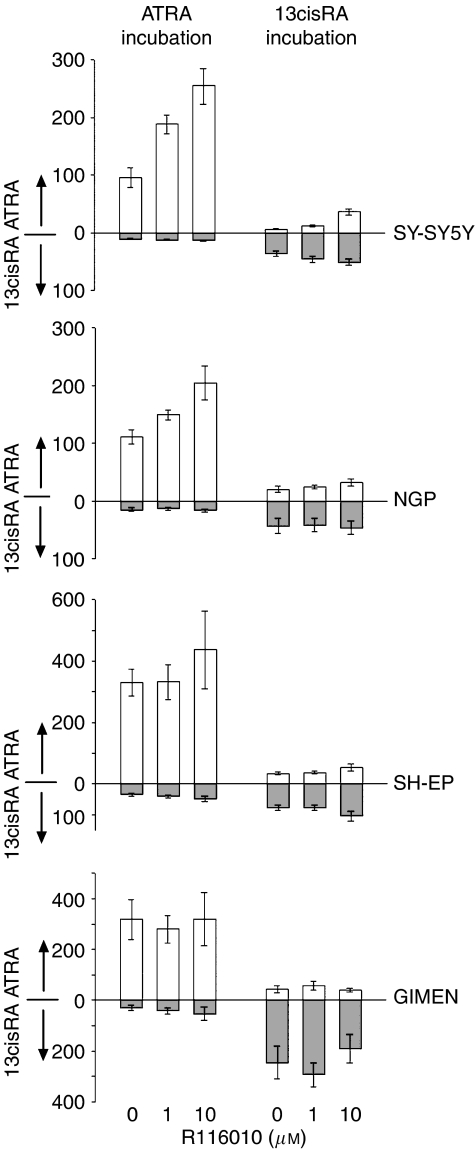
Effect of R116010 on ATRA metabolism in neuroblastoma cells. SH-SY5Y, NGP, SH-EP and GIMEN cells were treated with 10 *μ*M ATRA or 13cisRA in the absence or presence of 1 or 10 *μ*M R116010 for 24 h. Intracellular ATRA and 13cisRA levels were analysed by normal-phase HPLC. Values are expressed as concentrations based on cell counts and cell volume calculations, and data are mean values±s.e.m. (*n*⩾4).

We have previously suggested that R116010 can selectively inhibit ATRA metabolism in SH-SY5Y cells (Armstrong *et al*, 2005b). To test this hypothesis and investigate the effects on 13cisRA levels, the effect of R116010 was assessed in a panel of cell lines, which showed differing degrees of CYP26 isoform induction after RA treatment: two lines showed high levels of CYP26 induction (SH-SY5Y and NGP) and two showed low or undetectable CYP26 induction (SH-EP and GIMEN). After treatment of these cell lines with ATRA or 13cisRA alone (10 *μ*M) or in combination with R116010 (1 or 10 *μ*M) for 24 h there was a difference in relative levels of isomerisation between the ATRA and 13cisRA treatments, with ATRA accounting for approximately 15–31% of the total RA after 13cisRA incubation, compared to 13cisRA accounting for <10% of total RA after incubation with ATRA (Figure 3). For analysis, GLMs were used to compare RA isomer concentrations (ATRA and 13cisRA) with R116010 dose and cell type as fixed effects. Linear contrasts on R116010 dose were used to verify log dose-dependent effects, either on individual cell lines or the high/low CYP26 groups (where there were no significant difference between cell lines within the group) and incorporating zero dose as 1 log below the 1 *μ*M R116010 concentration. Incubation with R116010 resulted in significant increases in intracellular ATRA concentrations after treatment with ATRA in both SH-SY5Y and NGP cells (linear contrasts: F_1,26_=30.26, *P*<0.00001), but there was no effect of R116010 on ATRA concentrations in SH-EP or GIMEN cells (F_1,21_=0.017, *P*<0.9). Conversely, there was no effect of R116010 on 13cisRA levels after incubation with ATRA for any of the cell lines (F_2,55_=1.24, *P*=0.3). With respect to incubation with 13cisRA, SH-SY5Y cells exhibited a significant increase in ATRA concentrations after treatment in combination with R116010 (linear contrast, SH-SY5Y cells: F_1,15_=76.07, *P*<0.00001) and there was a similar, although nonsignificant, trend in NGP cells (linear contrast: F_1,12_=3.19, *P*=0.1). However, there was no observable effect of R116010 on ATRA levels after incubation with 13cisRA in SH-EP or GIMEN cells (low CYP26 group, F_2,28_=0.29, *P*>0.7). The effect of R116010 was again specific to ATRA levels, as there were no significant changes in 13cisRA concentrations after incubation with R116010 (GLM, effect of R116010, F_2,57_=0.256, *P*>0.7). Clearly, the activity of R116010 was limited to increasing the intracellular concentrations of ATRA only in the cell lines in which CYP26 was inducible to a high level.

**Figure 4:**
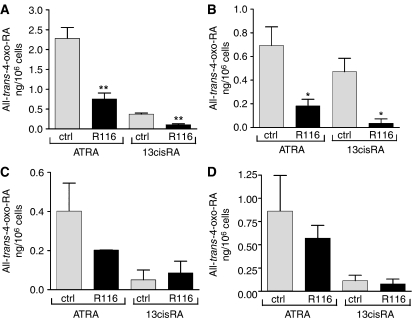
Effect of R116010 on ATRA metabolism in neuroblastoma cells. SH-SY5Y (**A**), NGP (**B**), SH-EP (**C**) and GIMEN (**D**) cells were treated with 10 *μ*M ATRA or 13cisRA in the absence or presence of 10 *μ*M R116010 for 24 h. Intracellular all-*trans*-4-oxo-RA levels were analysed by LC/MS. Values are expressed as ng 10^−6^ cells and are mean values±s.e.m. (*n*⩾4). Statistical significance relative to control cells is indicated by ^*^P<0.05, ^**^P<0.01.

To analyse the effect of R116010 on RA metabolism, LC/MS was used to evaluate levels of retinoid metabolites in these cells. For the high CYP26 expressing cells incubated with 10 *μ*M ATRA, there were significant reductions in intracellular AT-4-oxo-RA levels when co-incubated with R116010 (two-way ANOVA, effect of R116010: F_1,17_=18.9, *P*=0.00043; Figure 4) ranging from 3- to 3.8-fold for SH-SY5Y and NGP cells, respectively. Conversely, for the low CYP26-expressing cell lines incubated with 10 *μ*M ATRA there was no significant effect of R116010 (F_1,13_=0.68, *P*>0.4). R116010 also decreased AT-4-oxo-RA levels 3.5-fold in SH-SY5Y and 13.2-fold in NGP cells incubated with 13cisRA (F_1,18_=32.15, *P*=0.000022). Again, there was no significant effect in SH-EP and GIMEN cells (F_1,13_=0.02, *P*>0.8) under similar conditions (Figure 4).

### Effect of CYP26A1 and B1 siRNA on ATRA metabolism

**Figure 5:**
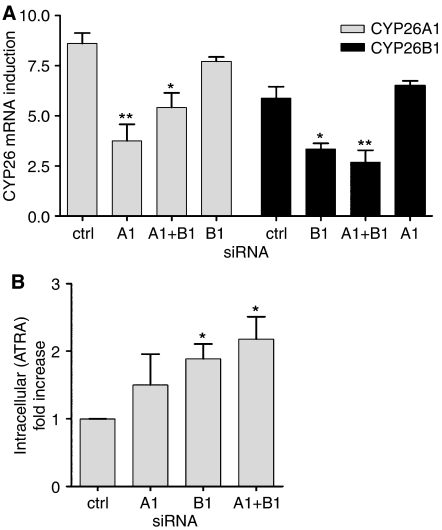
Effect of siRNA to CYP26 on ATRA metabolism. SH-SY5Y cells were transfected with psi-CYP26A1 (**A**1), psi-CYP26B1 (**B**1) either singly or in combination (A1+B1), or with psi-Ctrl (ctrl). 6 h after transfection, cells were treated with 0.1 *μ*M ATRA (**A**) or 10 *μ*M (**B**) for 40 h. In (**A**) reverse-transcribed RNA was subjected to real-time PCR using TaqMan probes for CYP26A1 (grey bars), CYP26B1 (black bars) and *β*-actin. Values are normalised for *β*-actin levels and expressed as fold increase relative to expression of CYP26 in SH-SY5Y cells treated with 0.01 *μ*M ATRA. Data are mean values±s.e.m. (*n*=3). In (**B**) intracellular ATRA concentrations were analysed by normal-phase HPLC. Values are expressed as fold increases in ATRA concentrations based on cell counts and cell volume calculations. Data are mean values±s.e.m. (*n*=3). Statistical significance relative to control cells is indicated by ^*^P<0.05, ^**^P<0.01.

To investigate the relationship between CYP26 and ATRA metabolism, RNA interference experiments were performed in SH-SY5Y cells to downregulate CYP26A1 and B1. SiRNA to CYP26A1 inhibited ATRA-induced (0.1 *μ*M) CYP26A1 mRNA expression by approximately 56%, while siRNA to CYP26B1 inhibited ATRA-induced CYP26B1 mRNA expression by approximately 43% (Figure 5A). These effects were specific for each CYP26 isoform, as siRNA to CYP26A1 did not significantly inhibit CYP26B1 mRNA expression, and vice versa. Transfection of CYP26A1 and B1 siRNA together resulted in comparable downregulation of each isoform relative to the individual siRNAs, with approximately 37 and 54% inhibition of CYP26 A1 and B1, respectively. The corresponding analysis of CYP26 protein expression was not possible due to a lack of suitable isoform-specific commercially available antibodies.

The effect of CYP26 siRNA on intracellular ATRA concentrations after ATRA treatment (10 *μ*M, 40 h) is shown in Figure 5B. ATRA concentrations increased by 1.5-fold (relative to untreated controls, not significant) in cells transfected with CYP26A1 siRNA, by 1.9-fold (*P*=0.016) in cells transfected with CYP26B1 siRNA, and by 2.2-fold (*P*=0.023) in cells transfected with both CYP26A1 and B1 siRNA. There were no significant increases in 13cisRA concentrations under these conditions. These data indicate that CYP26B1, and to a lesser extent CYP26A1, contribute to ATRA metabolism in neuroblastoma cells.

### Pharmacokinetic analysis of RA in an SH-SY5Y xenograft model

To determine the effect of R116010 on RA metabolism *in vivo*, we first analysed retinoid concentrations after treatment of CD-1 mice bearing SH-SY5Y xenografts with either ATRA or 13cisRA at 100 mg kg^−1^ for 0.5, 2 or 6 h (Table 1). Peak plasma levels were achieved after 2 h, and declined over the next 4 h, with increasing concentrations of the equivalent 4-oxo-RA metabolite. 13cisRA and 13cis-4-oxo-RA, or ATRA and AT-4-oxo-RA, accounted for <10% of total retinoids at all time points measured after treatment with ATRA or 13cisRA, respectively. Intratumoral concentrations of RA isomers and metabolites closely reflected those in plasma after treatment with ATRA, with ATRA accounting for ⩾80% of total retinoids at all time points. In comparison, after treatment with 13cisRA, intratumoral levels of 13cisRA decreased from 90 to 49% of total retinoids over 6 h, while intratumoral levels of ATRA, 13cis-4-oxo-RA and AT-4-oxo-RA increased to comparable levels at 6 h.

There was a significant shift from parent drug to the corresponding 4-oxo metabolite in liver samples after ATRA or 13cisRA treatment. Peak AT-4-oxo-RA levels (2.39 *μ*g g^−1^) at 6 h post-ATRA treatment accounted for 45% of total retinoids. An even greater extent of metabolism was observed after treatment with 13cisRA, with the peak 13cis-4-oxo-RA concentration (3.79 *μ*g g^−1^) accounting for 69% of total retinoids after 6 h. These data indicate that substantial metabolism of ATRA or 13cisRA occurred after 2 h, and as such, subsequent combination treatments with R116010 were performed for 6 h to evaluate its effect on RA metabolism.

### Effect of R116010 on retinoid concentrations in an SH-SY5Y xenograft model

**Figure 6:**
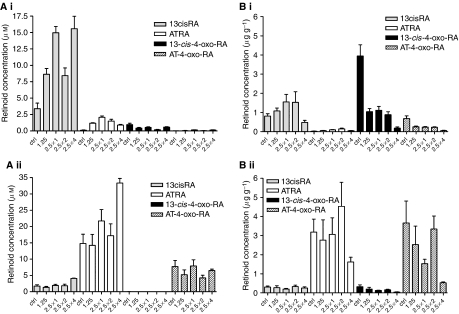
Effect of increasing doses of R116010 on plasma (**A**) and liver (**B**) retinoid levels in an SH-SY5Y xenograft model. CD-1 nude mice-bearing SH-SY5Y tumours were treated with 13cisRA (i) or ATRA (ii) (100 mg kg^−1^), either without or in combination with R116010 at 1.25 mg kg^−1^ (1.25), or with one or more doses of R116010 at 2.5 mg kg^−1^ (2.5 × 1, 2.5 × 2, 2.5 × 4). Animals were sacrificed and samples taken 6 h after dosing with retinoid. Plasma and liver retinoid concentrations were analysed by LC/MS. Data are mean values±s.e.m. (*n*⩾3).

Treatment of mice-bearing SH-SY5Y xenografts with increasing doses of R116010 in combination with 13cisRA resulted in significant increases in both 13cisRA and ATRA plasma concentrations compared to control animals treated with retinoid alone (ANOVA F_4,15_=13.91, *P*=0.00006 and F_4,15_=59.3, *P*<0.00001, respectively; Figure 6Ai). This effect was dependent on the dose of R116010 (linear contrasts F_1,15_=17.99, *P*=0.0007 and F_1,13_=22.5, *P*=0.0004), with up to a 4.6-fold increase in 13cisRA plasma concentration, compared to treatment with 13cisRA alone, after pretreatment with four doses of R116010 at 2.5 mg kg^−1^ (Figure 6Ai). There were also small but significant variations in the plasma concentrations of 13cis-4-oxo-RA and AT-4-oxo-RA after treatment with 13cisRA in combination with increasing doses of R116010 at 2.5 mg kg^−1^ compared to 13cisRA alone (ANOVA F_4,15_=7.15, *P*=0.002 and F_4,15_=3.71, *P*=0.027, respectively). However, for these metabolites there was either no significant dose-dependent effect (13cis-4-oxo-RA, linear contrast, F_1,15_=1.25, *P*=0.28), or the effect was an apparent linear increase in metabolite rather than the predicted decrease (linear contrast, AT-4-oxo-RA, F_1,15_=5.85, *P*=0.029). Similar although less marked results were obtained with respect to ATRA plasma concentrations in ATRA-treated mice, with a maximum 2.3-fold increase in plasma ATRA concentrations after pretreatment with four doses of R116010 at 2.5 mg kg^−1^ (ANOVA F_4,14_=3.43, *P*=0.037, linear contrast F_1,14_=11.0, *P*=0.0051; Figure 6Aii). The effect on plasma 13cisRA concentrations was marginal (ANOVA, F_4,14_=3.09, *P*=0.051). In these experiments, plasma 13cis-4-oxo-RA was not detectable and there was no effect on plasma AT-4-oxo-RA levels (ANOVA, F_1,14_=0.05, *P*>0.9).

Treatment of mice bearing SH-SY5Y xenografts with R116010 in combination with 13cisRA resulted in only a marginal apparent effect on tumour 13cisRA levels compared to 13cisRA alone (ANOVA F_3,10_=3.42, *P*=0.06) with only the single dose treatment with R116010 at 2.5 mg kg^−1^ producing a significant increase in tumour 13cisRA levels compared to control animals (0.22±0.04 to 0.59±0.07 *μ*g g^−1^, Dunnett's test *P*=0.046). Levels of ATRA in tumour samples from 13cisRA-treated mice were also not significantly affected by R116010 treatment (F_3,10_=0.69, *P*=0.58). However, CYP26A1 expression in tumours from animals treated with 13cisRA in combination with two doses of R116010 at 2.5 mg kg^−1^ increased 4- to 20-fold in individual animals, compared to CYP26A1 expression after treatment with 13cisRA alone (data not shown). There were no significant differences in levels of retinoids measured in tumour tissue after treatment of animals with R116010 before ATRA and no significant differences in levels of the 4-oxo-RA metabolites after treatment with either 13cisRA or ATRA, in combination with R116010 compared to 13cisRA or ATRA alone (ANOVA, F_3,10_<1.02, *P*>0.4). Furthermore, CYP26A1 expression was not significantly different in tumours from animals treated with R116010 prior ATRA.

In contrast to the results for tumour tissue, treatment of mice with R116010 before 13cisRA had a marked dose-dependent effect on concentrations of 13cis-4-oxo-RA and AT-4-oxo-RA in the liver (ANOVA, F_4,15_>15.18, *P*<0.0004, linear contrasts F_1,15_>50.28, *P*<0.00001; Figure 6Bi), giving significantly lower levels of both 13cis-4-oxo-RA and AT-4-oxo-RA under all conditions. These decreases in metabolite concentrations in hepatic tissue were not associated with 13cisRA levels being different from control (no R116010) animals (F_4,15_=3.32, *P*=0.039, Dunnett's tests not significant), although liver ATRA levels were increased slightly, with one and two doses of 2.5 mg kg^−1^ R116010 (ANOVA, F_4,13_=5.42, *P*=0.009; Dunnett's test, *P*⩽0.044; linear effect not significant, *P*=0.55). Treatment with ATRA in combination with R116010, or after R116010 pretreatment, resulted in significant decreases in levels of both 13cis-4-oxo-RA and AT-4-oxo-RA (ANOVA, F_4,14_>5.58, *P*<0.007, linear contrasts F_1,14_>17.9, *P*<0.0009; Figure 6Bii). No significant increases in liver 13cisRA or ATRA levels were observed after treatment of animals with ATRA in combination with R116010 (ANOVA, F_4,14_ <1.44, *P*>0.27).

## DISCUSSION

For the treatment of high-risk neuroblastoma, 13cisRA is administered on an intermittent schedule to minimise metabolism and maintain therapeutic concentrations. Our understanding of how RA works at a molecular level suggests that 13cisRA acts as a pro-drug, generating ATRA, the predominant biologically-active retinoid, by isomerisation. Strategies to increase activity of the ATRA isomer are, therefore, becoming increasingly important (Armstrong *et al*, 2005a). In this study, we observed a preferential uptake of ATRA compared to 13cisRA in all four cell lines, alongside a significant shift in RA isomerisation between 13cisRA and ATRA, after treatment of cells with 13cisRA compared to ATRA. R116010 selectively inhibited the metabolism of ATRA, with the magnitude of the R116010 effect proportional to the level of CYP26 induction in the cell lines. In addition, co-treatment of SH-SY5Y or NGP cells with R116010 and either ATRA or 13cisRA, compared to RA alone, resulted in an increased response in terms of expression of a panel of well-characterised retinoid responsive genes, consistent with the increase in intracellular ATRA concentrations. siRNA-mediated downregulation of CYP26 isoform expression in SH-SY5Y cells demonstrated the involvement of CYP26B1 in ATRA metabolism, although the contribution of CYP26A1 was less clear. Together, these data suggest that RA-inducible CYP26 is responsible for ATRA metabolism in neuroblastoma cells.

Preliminary time course experiments in an SH-SY5Y xenograft model demonstrated that ATRA or 13cisRA were the principle retinoids in the plasma and tumour fractions after treatment with ATRA or 13cisRA respectively. There was significant isomerisation of 13cisRA to ATRA in tumour samples after 13cisRA treatment, with ATRA accounting for 16% total retinoids after 6 h, despite ATRA accounting for only 1% total plasma retinoids under these conditions. This is in agreement with previous *in vitro* and *in vivo* data (Conley *et al*, 1999; Veal *et al*, 2002), and may represent either a superior uptake of ATRA compared to 13cisRA into the tumour, or increased intracellular isomerisation of 13cisRA to the more thermodynamically stable ATRA isomer.

The greatest effect of R116010 *in vivo* was observed when co-administrated with 13cisRA. This resulted in significant increases in plasma 13cisRA concentrations compared to treatment with 13cisRA alone. Moreover, significant decreases in liver 13cis-4-oxo-RA concentrations were seen under these conditions, while parent 13cisRA levels remained unchanged, indicative of an appreciably reduced metabolism of 13cisRA. As a consequence of higher 13cisRA levels, there were also higher levels of ATRA in the plasma and livers of animals treated with 13cisRA and R116010 compared to 13cisRA alone. Furthermore, the induction of CYP26A1 mRNA in SH-SY5Y tumours in response to 13cisRA was potentiated by co-treatment with R116010. However, an effect on ATRA metabolism was detected only when ATRA was administered with the highest dose of R116010. These results raise questions concerning the selectivity of R116010, and imply that the anticancer activity of R116010 may lie primarily in its effects on systemic RA metabolism.

There was evidence for considerable hepatic metabolism of 13cisRA compared to ATRA suggesting that these animals have the ability to catabolise 13cisRA more efficiently than ATRA. Furthermore, the greatest effect of R116010 was seen on 13cisRA metabolism. As CYP26 isoforms are specific for ATRA, this indicates that either CYP26 is not responsible for the R116010-regulatable activity in these animals, or that murine CYP26 is selective for 13cisRA. The selectivity of R116010 for CYP26 isoforms was determined by its inhibition profile towards other *P*450-mediated reactions in rat microsomes, including those enzymes known to metabolise 13cisRA (Van Heusden *et al*, 2002). However, this does not rule out selectivity of R116010 towards murine *P*450 homologues. The identification of the murine *P*450 CYP2C39 as an RA 4-hydroxylase (Andreola *et al*, 2004) demonstrates the existence of murine-specific *P*450 enzymes capable of metabolising RA, which in turn may be selective for 13cisRA and be inhibited by R116010. Whether the murine animal model is appropriate to study the effects of future compounds capable of inhibiting ATRA metabolism is clearly an important issue, which needs to be considered in future studies. The observation that treatment of NGP cells with R116010 alone resulted in increased expression of the MYCN oncogene *in vitro* highlights the need to study MYCN-amplified cell lines *in vivo,* and questions the benefit of R116010 as a single agent in this context.

Nevertheless, the effect of R116010 on plasma retinoid concentrations demonstrates that RAMBAs can inhibit systemic RA metabolism *in vivo,* which is likely to be dependent on hepatic CYP26 expression. Clinically, systemic inhibition of 13cisRA metabolism may be beneficial in neuroblastoma patients, providing a reservoir of 13cisRA for isomerisation to ATRA. This may be particularly advantageous in patients who have lower drug exposures, with extensive metabolism of 13cisRA and suboptimal oral administration both recently reported (Veal *et al*, 2007). There is considerable potential for RAMBAs to become part of 13cisRA treatment in neuroblastoma and this is an attractive target for future drug development. In this respect, it is essential that the most appropriate animal models are identified and utilised in future studies.

## Figures and Tables

**Table 1:** Pharmacokinetic analysis of RA in an SH-SY5Y xenograft model

**Tissue**	**RA isomer**	**0.5 h**	**% Total retinoids**	**2 h**	**% Total retinoids**	**6 h**	**% Total retinoids**
*ATRA*							
Plasma (*μ*M)	13cisRA	0.67±0.55	5	1.55±0.33	6	1.53±0.66	8
	ATRA	13.0±10.41	91	24.44±1.94	88	12.83±4.61	70
	13cis-4-oxoRA	0.03±0.01	<1	0.02±0.01	<1	0.05±0.03	<1
	AT-4-oxoRA	0.6±0.4	4	1.7±0.3	6	4.0±2.4	22
							
Liver (*μ*g g^−1^)^a^	13cisRA	0.42±0.3	11	0.40±0.08	5	0.25±0.06	5
	ATRA	3.07±1.95	77	6.92±1.13	81	2.40±0.36	46
	13cis-4-oxoRA	0.06±0.03	1	0.09±0.02	1	0.22±0.09	4
	AT-4-oxoRA	0.44±0.12	11	1.07±0.05	13	2.39±0.72	45
							
Tumour (*μ*g g^−1^)^a^	13cisRA	0.07±0.07	2	0.28±0.07	3	0.15±0.03	3
	ATRA	2.59±2.28	90	9.41±2.23	95	3.78±0.59	80
	13cis-4-oxoRA	0	0	0	0	0	0
	AT-4-oxoRA	0.21±0.09	8	0.24±0.06	2	0.79±0.38	17
							
*13cisRA*							
Plasma (*μ*M)	13cisRA	14.19±4.71	93	19.11±3.26	93	4.69±0.52	78
	ATRA	0.69±0.49	5	0.57±0.57	3	0.08±0.08	1
	13cis-4-oxoRA	0.27±0.11	2	0.63±0.10	3	1.22±0.24	20
	AT-4-oxoRA	0	0	0.13±0.04	<1	0	0
							
Liver (*μ*g g^−1^)^a^	13cisRA	3.77±1.05	75	2.33±0.11	58	0.98±0.12	18
	ATRA	0.12±0.08	2	0.05±0.05	1	0.02±0.02	<1
	13cis-4-oxoRA	0.89±0.22	18	1.33±0.08	33	3.79±0.99	69
	AT-4-oxoRA	0.26±0.10	5	0.30±0.09	8	0.67±0.25	12
							
Tumour (*μ*g g^−1^)^a^	13cisRA	2.0±0.67	90	2.12±0.57	81	0.28±0.04	49
	ATRA	0.15±0.09	7	0.37±0.16	14	0.09±0.02	16
	13cis-4-oxoRA	0.06±0.02	3	0.04±0.0	2	0.11±0.05	19
	AT-4-oxoRA	0.02±0.0	<1	0.09±0.04	3	0.09±0.02	16

Abbreviations: ATRA, all-*trans*-retinoic acid; LC/MS, liquid chromatography/mass spectrometry; RA, Retinoic acid.

CD-1 nude mice bearing SH-SY5Y tumours were treated with ATRA or 13cisRA (100 mg kg^−1^). Animals were killed and samples taken 0.5, 5, or 6 h after dosing. Retinoid concentrations were analysed by LC/MS. Data are mean values±s.e.m. (*n*⩾3).

a Assume 1 g=1 ml.

## References

[bib1] Andreola F, Hayhurst GP, Luo G, Ferguson SS, J GF, Goldstein JA, De Luca LM (2004) Mouse liver CYP2C39 is a novel retinoic acid 4-hydroxylase. Its down-regulation offers a molecular basis for liver retinoid accumulation and fibrosis in aryl hydrocarbon receptor-null mice. J Biol Chem 279: 3434–3438 14623888 10.1074/jbc.M305832200

[bib2] Armstrong JL, Redfern CPF, Veal GJ (2005a) 13-cis retinoic acid and isomerisation in paediatric oncology – is changing shape the key to success? Biochem Pharmacol 69: 1299–1306 15826600 10.1016/j.bcp.2005.02.003

[bib3] Armstrong JL, Ruiz M, Boddy AV, Redfern CPF, Pearson ADJ, Veal GJ (2005b) Increasing the intracellular availability of all-*trans*-retinoic acid in neuroblastoma cells. Br J Cancer 92: 696–704 15714209 10.1038/sj.bjc.6602398 PMC2361877

[bib4] Blaner WS (2001) Cellular metabolism and actions of 13-*cis*-retinoic acid. J Am Acad Dermatol 45: S129–S135 11606944 10.1067/mjd.2001.113714

[bib5] Conley BA, Ramsland TS, Sentz DL, Wu S, Rosen DM, Wollman M, Eiseman JL (1999) Antitumor activity, distribution, and metabolism of 13-cis-retinoic acid as a single agent or in combination with tamoxifen in established human MCF-7 xenografts in mice. Cancer Chemother Pharmacol 43: 183–197 9923548 10.1007/s002800050883

[bib6] Heise R, Mey J, Neis MM, Marquardt Y, Joussen S, Ott H, Wiederholt T, Kurschat P, Megahed M, Bickers DR, Merk HF, Baron JM (2006) Skin retinoid concentrations are modulated by CYP26AI expression restricted to basal keratinocytes in normal human skin and differentiated 3D skin models. J Invest Dermatol 126: 2473–2480 16778795 10.1038/sj.jid.5700432

[bib7] Idres N, Marill J, Flexor MA, Chabot GG (2002) Activation of retinoic acid receptor-dependent transcription by all-*trans*-retinoic acid metabolites and isomers. J Biol Chem 277: 31491–31498 12070176 10.1074/jbc.M205016200

[bib8] Mangelsdorf DJ, Borgmeyer U, Heyman RA, Zhou JY, Ong ES, Oro AE, Kakizuka A, Evans RM (1992) Characterization of three RXR genes that mediate the action of 9-cis retinoic acid. Genes Dev 6: 329–344 1312497 10.1101/gad.6.3.329

[bib9] Marill J, Capron CC, Idres N, Chabot GG (2002) Human cytochrome *P*450s involved in the metabolism of 9-*cis*- and 13-*cis*-retinoic acids. Biochem Pharmacol 63: 933–943 11911845 10.1016/s0006-2952(01)00925-x

[bib10] Marill J, Cresteil T, Lanotte M, Chabot GG (2000) Identification of human cytochrome *P*450s involved in the formation of all-*trans*-retinoic acid principal metabolites. Mol Pharmacol 58: 1341–1348 11093772 10.1124/mol.58.6.1341

[bib11] Matthay KK, Villablanca JG, Seeger RC, Stram DO, Harris RE, Ramsay NK, Swift P, Shimada H, Black CT, Brodeur GM, Gerbing RB, Reynolds CP (1999) Treatment of high-risk neuroblastoma with intensive chemotherapy, radiotherapy, autologous bone marrow transplantation, and 13-cis-retinoic acid. Children's Cancer Group. N Engl J Med 341: 1165–1173 10519894 10.1056/NEJM199910143411601

[bib12] Muindi J, Frankel SR, Miller WH, Jakubowski A, Scheinberg DA, Young CW, Dmitrovsky E, Warrell RPJ (1992) Continuous treatment with all-*trans* retinoic acid causes a progressive reduction in plasma drug concentrations: implications for relapse and retinoid resistance in patients with acute promyelocytic leukemia. Blood 79: 299–303 1309668

[bib13] Njar VC, Gediya L, Purushottamachar P, Chopra P, Vasaitis TS, Khandelwal A, Mehta J, Huynh C, Belosay A, Patel J (2006) Retinoic acid metabolism blocking agents (RAMBAs) for treatment of cancer and dermatological diseases. Bioorg Med Chem 14: 4323–4340 16530416 10.1016/j.bmc.2006.02.041

[bib14] Njar VCO (2002) Cytochrome *P*450 retinoic acid 4-hydroxylase inhibitors: potential agents for cancer therapy. Mini Rev Med Chem 2: 261–269 12370067 10.2174/1389557023406223

[bib15] Ozpolat B, Mehta K, Lopez-Berestein G (2005) Regulation of a highly specific retinoic acid-4-hydroxylase (CYP26A1) enzyme and all-*trans*-retinoic acid metabolism in human intestinal, liver, endothelial, and acute promyelocytic leukemia cells. Leuk Lymphoma 46: 1497–1506 16194896 10.1080/10428190500174737

[bib16] Petkovich M, Brand NJ, Krust A, Chambon P (1987) A human retinoic acid receptor which belongs to the family of nuclear receptors. Nature 330: 444–450 2825025 10.1038/330444a0

[bib17] Ponthan F, Borgstrom P, Hassan M, Wassberg E, Redfern CPF, Kogner P (2001) The vitamin A analogues: 13-cis retinoic acid, 9-cis retinoic acid, and Ro 13-6307 inhibit neuroblastoma tumour growth *in vivo*. Med Pediatr Oncol 36: 127–131 11464864 10.1002/1096-911X(20010101)36:1<127::AID-MPO1030>3.0.CO;2-B

[bib18] Reynolds CP, Matthay KK, Villablanca JG, Maurer BJ (2003) Retinoid therapy of high-risk neuroblastoma. Cancer Lett 197: 182–192 10.1016/s0304-3835(03)00108-3 12880980

[bib19] Smith G, Ibbotson SH, Comrie MM, Dawe RS, Bryden A, Ferguson J, Wolf CR (2006) Regulation of cutaneous drug-metabolizing enzymes and cytoprotective gene expression by topical drugs in human skin *in vivo*. Br J Dermatol 155: 275–281 16882163 10.1111/j.1365-2133.2006.07317.x

[bib20] Taimi M, Helvig C, Wisniewski J, Ramshaw H, White J, Amad M, Korczak B, Petkovich M (2004) A novel human cytochrome *P*450, CYP26C1, involved in metabolism of 9-cis and all-*trans* isomers of retinoic acid. J Biol Chem 279: 77–85 14532297 10.1074/jbc.M308337200

[bib21] Tsukada M, Schroder M, Roos TC, Chandraratna RA, Reichert U, Merk HF, Orfanos CE, Zouboulis CC (2000) 13-cis retinoic acid exerts its specific activity on human sebocytes through selective intracellular isomerization to all-*trans* retinoic acid and binding to retinoid acid receptors. J Invest Dermatol 115: 321–327 10951254 10.1046/j.1523-1747.2000.00066.x

[bib22] Van Heusden J, Van Ginckel R, Bruwiere H, Moelans P, Janssen B, Floren W, van der Leede BJ, van Dun J, Sanz G, Venet M, Dillen L, Van Hove C, Willemsens G, Janicot M, Wouters W (2002) Inhibition of all-*trans*-retinoic acid metabolism by R116010 induces antitumour activity. Br J Cancer 86: 605–611 11870544 10.1038/sj.bjc.6600056 PMC2375285

[bib23] Veal GJ, Cole M, Errington J, Pearson ADJ, Foot ABM, Whyman G, Boddy AV (2007) Pharmacokinetics and metabolism of 13-*cis*-retinoic acid (isotretinoin) in children with high-risk neuroblastoma – a study of the United Kingdom Children's Cancer Study Group. Br J Cancer 96: 424–431 17224928 10.1038/sj.bjc.6603554 PMC2360017

[bib24] Veal GJ, Errington J, Redfern CPF, Pearson ADJ, Boddy AV (2002) Influence of isomerisation on the growth inhibitory effects and cellular activity of 13-*cis* and all-*trans* retinoic acid in neuroblastoma cells. Biochem Pharmacol 63: 207–215 11841795 10.1016/s0006-2952(01)00844-9

[bib25] Villablanca JG, Khan AA, Avramis VI, Seeger RC, Matthay KK, Ramsay NK, Reynolds CP (1995) Phase I trial of 13-cis-retinoic acid in children with neuroblastoma following bone marrow transplantation. J Clin Oncol 13: 894–901 7707116 10.1200/JCO.1995.13.4.894

[bib26] White JA, Beckett-Jones B, Guo YD, Dilworth FJ, Bonasoro J, Jones G, Petkovich M (1997) cDNA cloning of human retinoic acid-metabolizing enzyme (hP450RAI) identifies a novel family of cytochromes *P*450. J Biol Chem 272: 18538–18541 9228017 10.1074/jbc.272.30.18538

[bib27] White JA, Ramshaw H, Taimi M, Stangle W, Zhang A, Everingham S, Creighton S, Tam S-P, Jones G, Petkovich M (2000) Identification of the human cytochrome *P*450, P450RAI-2, which is predominantly expressed in the adult cerebellum and is responsible for all-*trans*-retinoic acid metabolism. Proc Natl Acad Sci USA 97: 6403–6408 10823918 10.1073/pnas.120161397 PMC18615

